# Improving Risk Stratification in pT3 Upper Tract Urothelial Carcinoma: A Focus on Invasion Patterns

**DOI:** 10.3390/cancers17060923

**Published:** 2025-03-08

**Authors:** Yung-Tai Chen, Hsiang-Ying Lee, Wen-Jeng Wu, Chih-Hung Lin, Yuan-Hong Jiang, Yu-Khun Lee, Kuan-Hsun Huang, Yao-Chou Tsai

**Affiliations:** 1 Department of Urology, Taiwan Adventist Hospital, Taipei 10556, Taiwan; urochen831@gmail.com; 2 Department of Urology, National Taiwan University Hospital, College of Medicine, National Taiwan University, Taipei 10051, Taiwan; 3 Department of Urology, Kaohsiung Medical University Hospital, Kaohsiung 80756, Taiwan; ashum1009@hotmail.com (H.-Y.L.); wejewu@kmu.edu.tw (W.-J.W.); 4 Department of Urology, School of Medicine, College of Medicine, Kaohsiung Medical University, Kaohsiung 80708, Taiwan; 5 Graduate Institute of Clinical Medicine, College of Medicine, Kaohsiung Medical University, Kaohsiung 80708, Taiwan; 6 Department of Pathology, Kaohsiung Municipal Hsiaokang Hospital, Kaohsiung 80756, Taiwan; chlathelas@gmail.com; 7 Department of Pathology, College of Medicine, Kaohsiung Medical University, Kaohsiung 80756, Taiwan; 8 Department of Urology, Hualien Tzu Chi Hospital, Buddhist Tzu Chi Medical Foundation and Tzu Chi University, Hualien 97002, Taiwan; redeemerhd@gmail.com (Y.-H.J.); leeyukhun@gmail.com (Y.-K.L.); 9 Division of Urology, Department of surgery, Dalin Tzuchi Hospital, The Buddhist Tzu Chi Medical Foundation, Chia Yi 62247, Taiwan; lqcat921@gmail.com; 10 Division of Urology, Department of Surgery, Taipei Tzu Chi Hospital, The Buddhist Medical Foundation, New Taipei City 23142, Taiwan; 11 School of Medicine, Buddhist Tzu Chi University, Hualien 97004, Taiwan

**Keywords:** pathological stage, nephroureterectomy, upper urinary tract urothelial cancer

## Abstract

**Purpose:** Existing T3 subclassifications for upper tract urothelial cancer (UTUC) are limited by heterogeneity and a primary focus on renal pelvis tumors. Our study aimed to propose a novel pT3 subclassification system specifically tailored to pT3 UTUC patients. **Materials and Methods:** This study analyzed 120 pT3 UTUC cases from a Taiwanese multicenter registry, using a standardized pathology report and a single pathologist for evaluation. **Results:** Univariate analysis revealed survival differences based on existing subclassifications. Multivariate analysis identified concurrent fat and parenchyma invasion as an independent predictor of worse overall survival (OS), cancer-specific survival (CSS), and disease-free survival (DFS). **Conclusions**: This study proposes a novel pT3 subclassification incorporating fat and parenchyma invasion, applicable to all UTUC sites. This subclassification may improve risk stratification, guide treatment decisions, and ultimately enhance patient outcomes.

## Simple Summary

This research introduces a refined method for categorizing pT3 upper tract urothelial carcinoma (UTUC), a type of cancer affecting the lining of the kidney and ureter. Traditional classifications have struggled to accurately predict patient outcomes due to inconsistencies and a focus primarily on renal pelvis tumors. This study, involving 120 patients from Taiwan, proposes a new system that considers the specific patterns of tumor invasion: whether the cancer has spread into the surrounding fat, the kidney tissue itself, or both. The key finding is that tumors invading both fat and kidney tissue significantly correlate with poorer survival rates. This dual invasion pattern was identified as an independent predictor of worse overall survival, cancer-specific survival, and disease-free survival. In essence, patients with this specific invasion pattern face a higher risk. This new classification system, applicable to all UTUC locations, aims to provide a more precise tool for doctors to assess patient risk and tailor treatment strategies. By improving risk stratification, clinicians can make more informed decisions, potentially leading to better patient outcomes and more effective cancer management.

## 1. Introduction

Pathologic T3 disease of upper tract urothelial carcinoma (UTUC) is frequently associated with unfavorable survival outcomes and a higher incidence of disease recurrence following radical nephroureterectomy (RNU) [1,2,3]. The established pT3 subclassification systems exhibit considerable diversity, emphasizing the need for a comprehensive pathological subclassification system for pT3 disease and only feasible for renal pelvis UTUC [4,5,6,7,8,9,10]. This diversity is evident in the one of most common used subclassification system, which involves microscopic parenchymal invasion (pT3a), as opposed to macroscopic invasion and/or sinus fat invasion. Additionally, there is a distinction between renal medulla invasion only (pT3a) and renal cortex invasion (pT3b). A less common classification includes parenchymal invasion only (pT3a) and sinus fat invasion only (pT3b), with some cases involving both parenchymal and sinus fat invasion. While all of these mentioned subclassification systems have demonstrated significant prognostic implications, there remains a lack of global consensus on the pT3 subclassification.

Locally advanced pT3 disease is a common occurrence in urothelial carcinoma and is often accompanied by several unfavorable risk factors. These factors include multi-focal invasion, which is not limited to the renal pelvis, as well as large tumor size, high-grade histology, lymphovascular invasion, and positive nodal disease, which collectively have a significant impact on the oncological outcomes of affected patients [11]. While pT3 disease involving both the ureter and renal pelvis is not uncommon in upper tract urothelial carcinoma (UTUC), previous subclassification systems have primarily focused on tumors within the renal pelvis. Furthermore, our preliminary analysis indicated a substantial coexistence of ureteral fat and renal parenchyma invasion in approximately 40% of pT3 patients in Taiwan [12,13]. Therefore, a universal subclassification feasible for both renal pelvis and ureter are mandatory for pT3 UTUC.

Given the high prevalence of coexisting ureteral fat and parenchymal disease, as well as the frequent occurrence of both renal pelvis and ureter urothelial cancers, there is a pressing need for a universal subclassification system for pT3 disease in UTUC. In response to this need, our study aimed to address this gap by conducting a multicenter cohort analysis, involving centrally reviewed pathological cases, to propose a novel pT3 subclassification system specifically tailored to pT3 UTUC patients.

## 2. Material and Methods

### 2.1. Data Source

This cohort study was based on the multicenter UTUC registry by Taiwan UTUC Collaboration Group in Taiwan. The study received institutional review board approval (IRB no.: 08-X-037 and 12-X-004). This study followed the reporting guidelines of STROBE Statement. In our retrospective review of patients who underwent radical nephroureterectomy (RNU) or segmental resection for UTUC, a total of 756 cases were assessed. Among them, 377 cases met the eligibility criteria for specimen review. Cases without a definitive surgical treatment (RNU or segmental resection) or lacking accessible full sets of pathology slides were excluded from the pathology review. Those initial local pathologist confirmed pT3 disease but rejected by the central review pathologist were excluded for final analysis. In addition, those central review confirmed pT3 disease with nodal involvement were also excluded. Eligible cases with a complete set of slides were sent for pathology review. The definition of a complete set of slides included all sections examined by the local institutional pathologist. Ultimately, 120 cases of centrally review confirmed pT3 disease were deemed eligible for final analysis.

The American Joint Committee on Cancer (AJCC) staging manual, which is widely used for cancer staging, defines pT3 stage as “tumor invades beyond muscularis into perinephric fat or renal parenchyma”. It has been controversial regarding the subclassification according to perinephric fat or parenchyma invasion [10].

### 2.2. Histological Evaluation

The histological evaluation of UTUC specimens was conducted using a standardized histological report format approved by the Taiwan Pathology Society, based on the AJCC TNM staging system and the principles of pathology management for urothelial cancer in NCCN guidelines. A single pathologist, who is the recommended consultant genitourinary pathologist of the Taiwan Society of Pathology, performed the evaluation. The median number of slides reviewed was 9, with an inter-quartile range of 8 to 12. The histological diagnosis and staging were based on the version 8 AJCC TNM staging system, and the histological grade was determined according to the 2015 WHO/ISUP recommendation grading system [14]. The diagnosis of UTUC histological subtypes was based on the WHO classification of tumors, and diagnostic criteria were described in the classification [15]. Additionally, the evaluation recorded tumor configuration, stromal invasion, lymphovascular invasion (LVI), perineural invasion (PNI), and surgical margin invasion, in addition to staging, tumor grading, and histological subtypes.

### 2.3. Follow-Up

To detect recurrence or progression, patients underwent a structured follow-up protocol. This included more frequent evaluations in the first year after surgery, followed by less frequent checks in subsequent years. Cross-sectional imaging (CT and/or MRI) was employed to assess recurrence/progression-free status, while cystoscopy specifically targeted bladder recurrence. The definition of recurrence or metastasis en-compassed local tumor recurrence, regional lymph node metastasis, or distant spread.

### 2.4. Statistical Analysis

Demographic and clinicopathological differences between groups were analyzed using Pearson’s Chi-square test with Bonferroni correction for categorical variables. Kaplan–Meier survival curves were generated and compared using stratified log-rank tests. Cox proportional hazards models, adjusted for potential confounders selected via backward regression (SLE = 0.05, SLS = 0.1), were used to assess treatment effects on survival. The final multivariate model, refined manually to a significance level of *p* < 0.05, included relevant clinicopathological variables, significant and non-significant covariates. All tests were two-tailed, with *p* < 0.05 indicating statistical significance. Analyses were performed using IBM SPSS version 22, adhering to the standard statistical reporting format of the Taiwan UTUC collaboration group [16,17,18,19,20].

## 3. Results

### 3.1. Study Cohort and Baseline Characteristics

In total, we identified 377 cases eligible for central pathology review. Among them, pT0-1 stage was confirmed by review pathologist in 180/377 cases, pT2 44/377 cases, pT3 127/377 cases, and pT4 26/377 cases (Supplementary Table S1). There were 7 confirmed pT3 disease having nodal involvement then excluded from final analysis. Of those with confirmed pT3 disease, there were 21 patients with only renal parenchyma invasion, 61 patients with only fat invasion and 38 patients with both parenchyma and fat invasion. Here, we try to define these two histological findings as two distinct tumor aggressiveness behavior. The pT3 disease were categorized as pT3 with only perinephric/periureteral fat or only renal parenchyma invasion (T3 single group) and both fat and parenchyma invasion (T3 both group).

Table 1 summarized the baseline clinical characteristics of both groups. Both groups were comparable in all clinical and histological variables, except histological factor of lymphovascular invasion (LVI) and tumor size. The T3 both group was associated with more LVI than T3 single group (92.1% vs. 63.4%, *p* = 0.001). In addition, the T3 both group was associated with larger tumor size. The median follow-up periods were 40.4 months (IQR: 23.0–60.8) and 21.9 (IQR: 9.1–36.3) months, respectively.

### 3.2. Univariate Survival Analyses

In univariate analyses, survival difference was identified in OS (hazard ratio [HR], 2.722; 95% confidence interval [CI], 1.530–4.843; *p* = 0.001, CSS HR, 3.090; 95% CI, 1.389–6.871; *p* = 0.006) and DFS (HR, 2.074; 95% [CI], 1.218–3.533; *p* = 0.007) (Table 2). Both fat and parenchyma invasion, ECOG ≥ 1, involvement of both the ureter and renal pelvis, multiplicity, and perineural invasion (PNI) were common risk factors for OS, CSS, and DFS. Peri-operative adjuvant chemotherapy was the only protective factor for survival outcome (OS: HR, 0.343; 95% CI, 0.171–0.689; *p* = 0.003).

### 3.3. Multivariate Survival Analysis

In multivariate analysis, both fat and parenchyma invasion were the common independent risk factor for OS (HR, 3.570; 95% [CI], 1.926–6.617; *p* < 0.001, CSS (HR, 3.789; 95% [CI], 1.557–9.224; *p* = 0.003) and DFS (HR, 2.283; 95% [CI], 1.275–4.088; *p* = 0.005) (Table 3). The ECOG ≥ 1 was the common independent risk factors for OS CSS and DFS. Multiplicity of UTUC was the common independent risk factor for cancer-related death and disease progression. Peri-neural invasion was the common independent risk factor for OS and CSS. Peri-operative adjuvant chemotherapy was the only independent protective factor for OS (HR, 0.454; 95% [CI], 0.220–0.937; *p* = 0.033).

**Figure 1:**
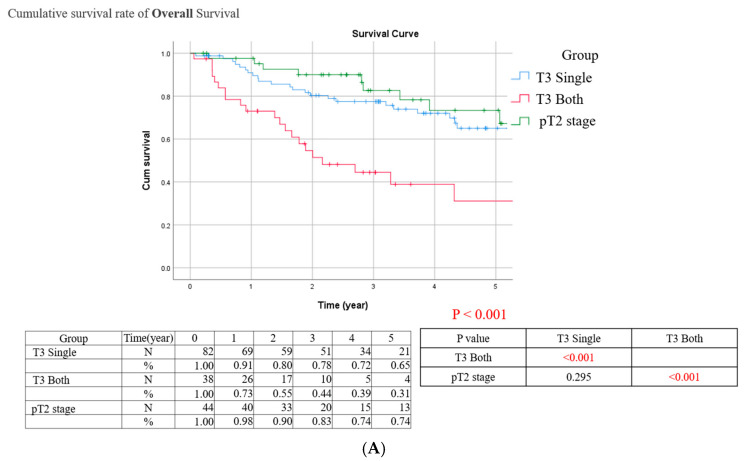

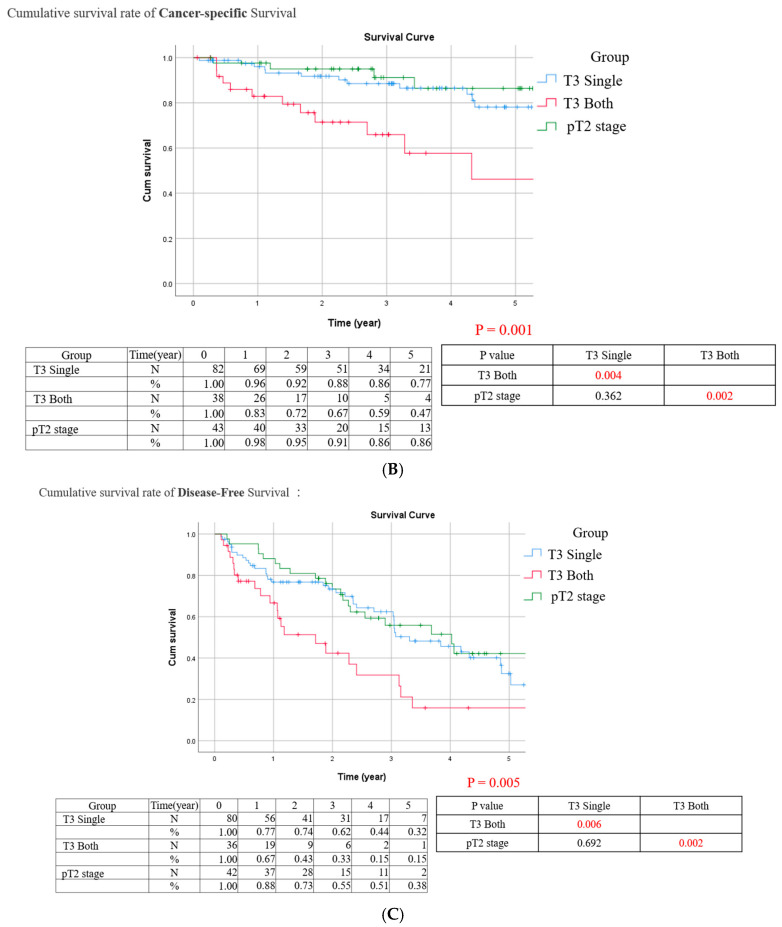
(**A**) Overall survival (OS), (**B**) cancer-specific survival (CSS), and (**C**) disease-free survival (DFS) Kaplan–Meier curves stratified by the novel pT3 subclassification system. The pT3 single group includes tumors invading either the renal parenchyma or the sinus fat, while the pT3 both group includes tumors invading both. The T2 group represents the pT2 stage tumors confirmed by central pathology. Log-rank test was used to compare survival curves between groups.

In the Kaplan–Meier survival curve, a sub-group of central pathology confirmed pT2 cases were enrolled for further comparison with T3 single and T3 both groups. A comparison of Kaplan–Meier estimated survival curves among the T2 group, the T3 single group and the T3 both groups revealed that the T3 both group was associated with statistically significant worse OS, CSS and DFS than T2 and T3 single groups (Figure 1). In addition, the T2 and T3 single groups had comparable OS, CSS, and DFS.

## 4. Discussion

In this retrospective multicenter cohort study comprising Taiwanese patients diagnosed with pT3 UTUC, the clinical characteristics and pathological findings were reviewed and subjected to centralized pathological review. Based on the observed pathological patterns and survival outcomes in the Taiwanese UTUC cohort, our novel pT3 subclassification helps identify patients who had both renal parenchyma and sinus fat invasion were high risk for overall mortality, cancer-related death, and disease progression. This novel subclassification will not be limited to patients with pure renal sinus fat/pure parenchyma invasion, moreover, also feasible in conditions with multifocal peri-ureteral fat plus renal parenchyma invasion. The results of current study will contribute to improved prognostication and treatment stratification in patients with pT3 UTUC, ultimately enhancing patient outcomes and guiding clinical decision-making.

The mechanisms underlying parenchyma invasion and sinus fat invasion in UTUC are likely to differ, suggesting potential variations in tumor cell invasiveness. In the case of parenchyma invasion, a range of cellular and molecular processes are often implicated, including modifications in cell adhesion, enhanced cell motility, secretion of matrix-degrading enzymes, and interactions with components of the extracellular matrix [21]. Conversely, sinus fat invasion is thought to be primarily driven by the tumor cells’ capacity to disrupt the architecture of adipose tissue and stimulate angiogenesis, which supports their growth and invasive behavior. As a result, these distinct invasiveness patterns in UTUC may reflect different abilities of tumor cells. Notably, UTUC cases demonstrating both parenchyma invasion and sinus fat invasion may indicate a higher level of disease aggressiveness. Therefore, our proposed subclassification primarily considers the additional invasiveness exhibited by tumor cells, rather than different tumor invasive capabilities.

Our findings are also supported by one retrospective multi-center study in Japan [5]. While the study provides valuable insights regarding the importance of pT3 subclassification, this study demonstrated a key distinction: pT3a tumors, defined by renal medulla invasion without sinus fat involvement, showed survival comparable to pT2. However, pT3b tumors with multi-site invasion (parenchyma and/or sinus fat) had significantly poorer survival in their central pathology reviewed cohort. This observation lends partial support to our novel pT3 subclassification, which suggests that UTUC with involvement of only one neighboring tissue has similar survival to pT2, while the study was limited by limited histology factors (pT and pN stage) being considered in their multivariable analysis without including significant clinical and histological factors. It is imperative to conduct additional extensive comparative cohort studies and long-term follow-ups to determine conclusively whether UTUC with parenchymal invasion should indeed be classified as true pT3 disease in future investigations.

Although the pT3 subclassification in various definitions had been confirmed in numerous scientific papers, it remained controversial whether replace this novel subclassification to current pT3 AJCC/UICC (Union for International Cancer Control) classification for UTUC [3,4,5,6,7,8,9,10]. One of the them was categorized as pT3a (microscopic infiltration of the renal parenchyma) or pT3b (macroscopic infiltration of the renal parenchyma and/or infiltration of peripelvic adipose tissue), which totally enrolled 284 cases from 11 centers without a central pathology review, potentially introducing inter-observer variability in the assessment of tumor stage and other important pathological features [4]. While the study provides valuable insights into the prognostic value of subclassification for pT3 UTUC, the number of cases of coexisting parenchyma and fat invasion were not reported among those with pT3b, which possibly mixed low-risk and high-risk patients in one subgroup. Additionally, this subclassification fail to identify patients at risk of cancer-related mortality and recurrence in multivariable analysis.

Unlike other studies, one recent Japanese retrospective cohort categorized pT3 disease as patients with renal parenchymal invasion only (pT3a) and those with peri-pelvic or periureteral fat invasion (pT3b) [10]. In their univariate analyses, patients with pT3b disease had a significantly lower 5-year DFS, 5-year CSS, and 5-year OS than those with pT3a disease. However, in multivariate analyses, the pT3b disease was not associated with survival outcomes. Moreover, this study is also limited by potential inter-observer variability due to non-centrally pathology review design.

Seisen and their colleagues categorized pT3 into patients with infiltration of the renal parenchyma on a microscopic level (pT3a) and those with infiltration of the renal parenchyma on gross inspection of the resection specimen and/or invasion of peripelvic fat (pT3b) [9]. This multicenter study was centrally pathology reviewed and inverse probability weighted (IPTW) to adjust potential confounders. In their IPW-adjusted Cox regression model, pT3b was associated with a significant risk for cancer-related death and disease recurrence (RFS [HR] = 2.02; *p* < 0.001) and CSS HR = 1.84; *p* = 0.005). However, the definition of the renal parenchyma invasion visible on gross inspection was not clearly defined, which limited the generalizability of the findings to other pathologists and institutes.

In our multivariate survival model for overall survival (OS), several factors emerged as independent predictors of worse outcomes: higher Eastern Cooperative Oncology Group (ECOG) performance status, advanced age, lack of adjuvant chemotherapy, and positive perineural invasion. Both high ECOG status and advanced age acted as competing risk factors for OS, likely reflecting underlying comorbidities and diminished physiological reserve [22]. Similarly, patients with upper tract urothelial carcinoma (UTUC) who did not receive adjuvant chemotherapy often presented with multiple comorbidities and poor general condition, further compounding their prognosis and acting as another competing risk factor for OS [23].

For cancer-specific survival (CSS), a higher ECOG status, tumor multiplicity, and perineural invasion were identified as independent risk factors. In locally advanced pT3 UTUC, multiplicity and perineural invasion may indicate multifocal and deep tumor infiltration, potentially translating to worse cancer-specific outcomes. A high ECOG status in UTUC patients is likely associated with poorer overall well-being and potentially compromised immune surveillance against micro-residual disease.

Finally, in the disease-free survival (DFS) model, a higher ECOG status and tumor multiplicity were independent risk factors. In locally advanced pT3 disease, multiplicity is often associated with multi-site deep tumor infiltration, potentially increasing the risk of residual disease post-radical surgery. A higher ECOG status may reflect impaired immunity against residual cancer cells, thereby increasing the likelihood of disease recurrence after surgery [24,25]. Finally, the observed lower incidence of intravesical recurrence in female patients after surgery may be linked to their typically lower cigarette exposure compared to male patients.

Locally advanced UTUC is commonly associated with a high risk of regional nodal involvement, which cases were commonly enrolled in prior pT3 subclassification studies [11]. Although nodal positive pT3 disease remained a locally advanced disease, this histological presentation was actually an independent risk factor for cancer recurrence as well as cancer-related death [11]. Therefore, aggressive systemic treatments were commonly recommended for their high risk of systemic spread of this locally advanced disease [11,26]. Clarifying the impact of pT3 subclassification without excluding cases [24] with nodal involvement clearly introduced a strong bias by enrolling significant pT3 cases already with systemic spread. In addition, survival models excluding cases with nodal involvement could help in predicting cases who required aggressive systemic therapy. Our study revealed that a compromised performance status, advanced age, tumor multiplicity, and positive peri-neural invasion independently contributed to adverse survival outcomes. Notably, post-operative adjuvant chemotherapy emerged as a factor associated with improved survival in cases of locally advanced UTUC.

We acknowledge that the initial inclusion of cases with ureteral lesion only, categorized as “pT3 single,” could potentially introduce heterogeneity. However, we included ureteral pT3 lesions for several key reasons, which we believe are important to consider in the context of the rarity and complexity of UTUC staging: firstly, while UTUC pT3 disease is often stratified by location (ureter vs. renal pelvis), the clinical significance of this distinction for survival outcomes has not been consistently demonstrated [27]. Existing location-based sub-classifications of pT3 have not been shown to reliably predict prognosis. Secondly, the current AJCC staging system for UTUC does not incorporate location-based subclassification within the pT3 category. Our study aimed to address the need for a more straightforward and clinically relevant pT3 staging system, regardless of tumor location. Thirdly, UTUC, especially locally advanced pT3 disease, is relatively rare. Excluding ureteral lesions would significantly reduce our sample size, potentially compromising the statistical power of our analysis and limiting our ability to draw meaningful conclusions. Fourthly, this study is intended to explore potential refinements to the pT3 staging system. To address this specific concern, we performed a sensitivity analysis excluding the 46 cases solely located in the ureter. This resulted in 37 patients in the “T3 single” group and 37 in the “T3 both” group. While the Kaplan–Meier survival curves still demonstrated significantly better overall survival and disease-free survival for the “T3 single” group (Supplementary Figures S1–S3), the difference in cancer-specific survival became less pronounced, although the curves still showed a trend towards better survival in the “T3 single” group. We speculate that the reduced sample size in this subgroup analysis might have limited the statistical power to detect a significant difference in CSS, potentially underestimating the true outcome difference.

Furthermore, we conducted an additional analysis comparing outcomes based on pT3 single involvement in the ureter (n = 45) versus the renal pelvis (n = 19). Kaplan–Meier survival analysis showed no significant difference in OS, CSS, or DFS between these two groups (Supplementary Figures S1–S3). These preliminary data suggest that location-specific T3 subclassification may not be clinically significant. Based on these findings, we speculate that the location of pT3 involvement (ureter vs. renal pelvis) may not be a critical determinant of prognosis. While our current data suggest this, we acknowledge the limitations of our retrospective study design and relatively small sample size. Therefore, we strongly believe that a prospective, large-scale study with central histological review is crucial to validate our speculation and definitively address the role of tumor location in UTUC staging. Such a study would be invaluable in refining the current AJCC staging system for UTUC and ultimately improving patient care.

## 5. Limitations

This study was limited by several factors. First, the current study is still limited by a small sample size, which limited the power of detecting risk factors for survival outcomes. Second, the retrospective nature of the study design is subjective to selection bias. Third, a lymphadenectomy was mainly performed in patients with clinical suspicious nodal diseases or advanced clinical stages; therefore, it was not routinely performed on RNU patients in Taiwan [18,19,28,29,30]. Fourth, the exclusion of pT3 cases with nodal involvement and those rejected by the central review pathologist could introduce selection bias. Finally, the proposed novel pT3 subclassification system was based on the Taiwanese UTUC cohort. The lack of external validation in other populations or geographic regions limits the generalizability of these findings.

## 6. Conclusions

This study explored the potential of a novel pT3 subclassification system for UTUC based on the pattern of renal parenchyma and perinephric fat invasion in Taiwanese patients. The findings suggest that this subclassification system holds promise for improved risk stratification within pT3 UTUC. Patients classified as having both fat and parenchymal invasion exhibited significantly worse overall survival, cancer-specific survival, and disease-free survival compared to those with invasion limited to either fat or parenchyma invasion only. Further research with larger and more diverse cohorts, prospective designs, and external validation is needed to confirm the findings and establish the clinical utility of the proposed subclassification system.

## Figures and Tables

**Table 1:** Baseline demographic data of the pT3 upper tract urothelial cancer patients undergoing radical nephroureterectomy and segmental resection.

z	T3 Single (N = 82)		T3 Both (N = 38)		*p*-Value ^a^
	N	%	N	%	
Gender					
men	38	(46.3)	18	(47.4)	0.916
women	44	(53.7)	20	(52.6)	
Age					
<50	3	(3.7)	1	(2.6)	0.842
50~70	36	(43.9)	15	(39.5)	
>70	43	(52.4)	22	(57.9)	
ECOG					
0	38	(46.3)	14	(36.8)	0.192
1	38	(46.3)	24	(63.2)	
2	3	(3.7)	0	(0.0)	
3	3	(3.7)	0	(0.0)	
Comorbidity					
Coronary artery disease	3	(3.7)	3	(7.9)	0.322
Arrythmia	5	(6.1)	4	(10.5)	0.392
Hypertension	41	(50.0)	24	(63.2)	0.178
End stage renal disease	5	(6.1)	6	(15.8)	0.087
Diabetes	17	(20.7)	12	(31.6)	0.197
Other Malignancy (not UTUC/bladder UC)	9	(11.0)	7	(18.4)	0.264
Laterality					
Left	35	(42.7)	22	(57.9)	0.081
Right	47	(57.3)	15	(39.5)	
Graft kidney	0	(0.0)	1	(2.6)	
Tumor location					
Renal pelvis	19	(23.2)	23	(60.5)	<0.001 **
Ureter	45	(54.9)	1	(2.6)	
Renal pelvis + Ureter	18	(22.0)	14	(36.8)	
Multiplicity					
No	48	(58.5)	19	(50.0)	0.381
Yes	34	(41.5)	19	(50.0)	
Tumor size					
<1 cm	3	(3.7)	0	(0.0)	0.001 **
≥1 and <2 cm	22	(26.8)	2	(5.3)	
≥2 and <3 cm	18	(22.0)	3	(7.9)	
≥3 cm	39	(47.6)	33	(86.8)	
Histological evaluation					
Grade (%)					
Low grade	1	(1.2)	0	(0)	1
High grade	81	(98.8)	38	(100)	
Tumor necrosis (%)					
No	78	(95.1)	34	(89.5)	0.447
Yes	4	(4.9)	4	(10.5)	
Perineural invasion (%)					
No	64	(78.0)	25	(65.8)	0.229
Yes	18	(22.0)	13	(34.2)	
Lymphovascular invasion (%)					
No	30	(36.6)	3	(7.9)	0.002 *
Yes	52	(63.4)	35	(92.1)	
Configuration (%)					
Papillary	24 (29.3)	24 (29.3)	12	(31.6)	0.776
non-papillary	40 (48.8)	40 (48.8)	16	(42.1)	
Mixed	18 (22.0)	18 (22.0)	10	(26.3)	
Multiplicity (%)					
No	48	(58.5)	19	(50.0)	0.498
Yes	34	(41.5)	19	(50.0)	
Carcinoma in situ (%)					
No	74	(90.2)	36	(94.7)	0.636
Yes	8	(9.8)	2	(5.3)	
Histological subtypes					
Small cell	2		1		N.A.
Mixed	5		1		
Poorly differentiated	6		1		
Sarcomatoid	3		5		
Grandular	1		1		
Giant cell	3		1		
Clear cell	2		1		
Nested	4		2		
Squamous	2		2		
Plasmacytoid	1		0		
Lymphoepithelioma	1		0		
Synchronous bladder tumor					
No	67	(81.7)	29	(76.3)	0.746
Previous Hx of bladder UC	6	(7.3)	3	(7.9)	
Concurrent Bladder UC	9	(11.0)	6	(15.8)	
Adjuvant Chemotherapy					
No	50	(61.0)	24	(63.2)	0.819
Yes	32	(39.0)	14	(36.8)	

^a^ Chi-Squared test calculated for the difference variables. * <0.05, ** <0.01.

**Table 2:** Comparative univariate survival analysis the pT3 upper tract urothelial cancer patients undergoing radical nephroureterectomy and segmental resection.

Univariate Analysis	OS		CSS		DFS		BRFS	
	HR (95% CI)	*p*-Value	HR (95% CI)	*p*-Value	HR (95% CI)	*p*-Value	HR (95% CI)	*p*-Value
Group								
T3 Single	1		1		1		1	
T3 Both	2.722 (1.530, 4.843)	0.001 **	3.090 (1.389, 6.871)	0.006 **	2.074 (1.218, 3.533)	0.007 **	1.668 (0.677, 4.108)	0.266
Group according to invasion								
Parenchyma only	1		1		1		1	
Peri-sinus/ureter fat only	1.127 (0.451, 2.814)	0.798	1.875 (0.414, 8.490)	0.415	1.179 (0.559, 2.486)	0.665	0.936 (0.298, 2.941)	0.910
Both parenchyma and fat	2.980 (1.199, 7.406)	0.019 *	5.110 (1.131, 23.096)	0.034 *	2.350 (1.073, 5.146)	0.033 *	1.589 (0.464, 5.439)	0.461
ECOG								
0	1		1		1		1	
1~3	2.745 (1.473, 5.114)	0.001 **	3.842 (1.517, 9.733)	0.005 **	2.701 (1.565, 4.661)	<0.001 **	1.312 (0.564, 3.054)	0.528
Age								
<70	1		1		1		1	
≥70	2.620 (1.416, 4.848)	0.002 *	2.457 (1.055, 5.722)	0.037 *	1.270 (0.765, 2.111)	0.356	1.652 (0.705, 3.867)	0.248
Sex								
Male	1		1		1		1	
Female	1.233 (0.697, 2.182)	0.471	1.212 (0.550, 2.671)	0.633	1.312 (0.787, 2.187)	0.298	0.194 (0.065, 0.572)	0.003 **
Tumor location								
Renal pelvis	1		1		1		1	
Ureter	0.806 (0.389, 1.672)	0.562	0.846 (0.273, 2.628)	0.773	0.788 (0.433, 1.434)	0.436	0.920 (0.333, 2.536)	0.871
Renal pelvis+ Ureter	2.258 (1.146, 4.450)	0.019 *	3.761 (1.427, 9.914)	0.007 **	1.896 (1.014, 3.546)	0.045 *	1.904 (0.667, 5.436)	0.229
Tumor size								
<3 cm	1		1		1		1	
≥3 cm	1.719 (0.933, 3.167)	0.083	1.664 (0.717, 3.863)	0.236	1.331 (0.791, 2.237)	0.281	1.372 (0.575, 3.274)	0.476
Multiplicity								
No	1		1		1		1	
Yes	2.241 (1.264, 3.971)	0.006 **	4.379 (1.824, 10.510)	0.001 **	1.917 (1.152, 3.190)	0.012 *	2.207 (0.951, 5.122)	0.065
Synchronous bladder tumor								
No	1		1		1		1	
Previous Hx of bladder UC	0.971 (0.298, 3.161)	0.961	0.000 (0.000, infinity)	0.982	0.725 (0.225, 2.337)	0.591	3.453 (0.955, 12.489)	0.059
Concurrent Bladder UC	1.909 (0.915, 3.985)	0.085	3.023 (1.252, 7.299)	0.014 *	1.700 (0.858, 3.368)	0.128	5.634 (2.260, 14.049)	<0.001 **
Adjuvant chemotherapy								
No	1		1		1		1	
Yes	0.343 (0.171, 0.689)	0.003 **	0.519 (0.216, 1.244)	0.142	0.852 (0.513, 1.415)	0.535	0.547 (0.222, 1.345)	0.188
Tumor Necrosis								
No	1		1		1		1	
Yes	1.189 (0.369, 3.829)	0.771	2.350 (0.703, 7.856)	0.165	1.660 (0.662, 4.161)	0.280	0.929 (0.125, 6.914)	0.942
CIS								
No	1		1		1		1	
Yes	1.276 (0.504, 3.226)	0.607	0.931 (0.219, 3.950)	0.922	2.083 (0.944, 4.598)	0.069	0.433 (0.058, 3.224)	0.414
Coronary artery disease								
No	1		1		1		1	
Yes	1.627 (0.582, 4.546)	0.353	1.487 (0.348, 6.344)	0.592	0.650 (0.158, 2.667)	0.550	1.337 (0.180, 9.951)	0.777
Arrythmia								
No	1		1		1		1	
Yes	1.122 (0.403, 3.127)	0.825	0.536 (0.072, 3.968)	0.542	0.833 (0.302, 2.299)	0.725	0.678 (0.091, 5.046)	0.704
End stage renal disease								
No	1		1		1		1	
Yes	1.002 (0.359, 2.793)	0.997	0.474 (0.064, 3.505)	0.464	0.381 (0.093, 1.562)	0.180	0.549 (0.074, 4.082)	0.558
Hypertension								
No	1		1		1		1	
Yes	1.035 (0.587, 1.824)	0.905	0.866 (0.395, 1.899)	0.720	1.080 (0.653, 1.789)	0.763	1.807 (0.757, 4.311)	0.182
Diabetes								
No	1		1		1		1	
Yes	1.682 (0.900, 3.146)	0.103	2.674 (1.195, 5.982)	0.017 *	1.404 (0.792, 2.486)	0.245	1.853 (0.754, 4.553)	0.179
Other Malignancy								
No	1		1		1		1	
Yes	1.014 (0.430, 2.393)	0.974	0.302 (0.041, 2.240)	0.242	0.811 (0.369, 1.784)	0.603	1.282 (0.434, 3.791)	0.653
Grade								
Low grade	1		1		1		1	
High grade	0.132 (0.017, 1.004)	0.050	20.365 (0.000, infinity)	0.865	0.246 (0.033, 1.820)	0.170	20.406 (0.000, infinity)	0.847
Configuration								
Papillary	1		1		1		1	
non-papillary	0.820 (0.422, 1.591)	0.557	0.583 (0.231, 1.470)	0.253	0.793 (0.450, 1.398)	0.423	0.473 (0.182, 1.228)	0.124
mixed	0.764 (0.356, 1.638)	0.489	0.731 (0.271, 1.973)	0.537	0.546 (0.268, 1.112)	0.096	0.553 (0.185, 1.653)	0.289
Lymphovascular invasion								
Negative/free	1		1		1		1	
Positive	1.977 (0.980, 3.989)	0.057	2.695 (0.920, 7.893)	0.071	2.100 (1.135, 3.884)	0.018 *	1.065 (0.434, 2.615)	0.890
Peri-neural invasion								
Negative/free	1		1		1		1	
Positive	2.565 (1.423, 4.623)	0.002 **	3.442 (1.550, 7.641)	0.002 **	2.163 (1.251, 3.740)	0.006 **	1.290 (0.475, 3.506)	0.617
Margins								
Free	1		1		1		1	
Not free	1.717 (0.843, 3.496)	0.136	2.470 (1.014, 6.018)	0.047 *	1.897 (0.970, 3.709)	0.061	1.911 (0.695, 5.251)	0.209
Pure urothelial carcinoma								
No	1		1		1		1	
Yes	0.667 (0.372, 1.198)	0.175	0.745 (0.335, 1.660)	0.472	0.805 (0.486, 1.335)	0.401	1.268 (0.548, 2.937)	0.579

OS, overall survival; CSS, cancer-specific survival; DFS, disease-free survival; BRFS, bladder recurrence-free survival. * <0.05, ** <0.01.

**Table 3:** Comparative multivariable survival analysis of pT3 upper tract urothelial cancer (UC) patients undergoing radical nephroureterectomy and segmental resection.

Multivariable Analysis	OS		CSS		DFS		BRFS	
	HR (95% CI)	*p*-Value	HR (95% CI)	*p*-Value	HR (95% CI)	*p*-Value	HR (95% CI)	*p*-Value
Group								
T3 Single	1		1		1		1	
T3 Both	3.570 (1.926, 6.617)	<0.001 *	3.789 (1.557, 9.224)	0.003 **	2.283 (1.275, 4.088)	0.005 **	1.932 (0.770, 4.848)	0.160
ECOG								
0	1		1		1			
1~3	2.022 (1.075, 3.804)	0.029 *	3.159 (1.217, 8.200)	0.018 *	2.620 (1.499, 4.582)	0.001 **		
Age								
<70	1		1				1	
>70	2.328 (1.224, 4.430)	0.010	2.186 (0.925, 5.169)	0.075			2.585 (1.052, 6.351)	0.038 *
Sex								
Male							1	
Female							0.189 (0.059, 0.599)	0.005 **
Multiplicity								
No			1		1			
Yes			3.058 (1.240, 7.542)	0.015 *	2.056 (1.225, 3.451)	0.006 **		
Synchronous bladder tumor								
No							1	
Prior bladder UC							1.480 (0.382, 5.740)	0.571
Concurrent bladder UC							3.882 (1.519, 9.923)	0.005 **
Adjuvant chemotherapy								
No	1							
Yes	0.454 (0.220, 0.937)	0.033 *						
Lymphovascular invasion								
Negative					1			
Positive					1.775 (0.941, 3.349)	0.077		
Peri-neural invasion								
Negative	1		1					
Positive	2.254 (1.232, 4.125)	0.008 *	2.333 (1.006, 5.410)	0.048 *				

OS, overall survival; CSS, cancer-specific survival; DFS, disease-free survival; BRFS, bladder recurrence-free survival. * <0.05, ** <0.01.

## Data Availability

The datasets used and/or analyzed during the current study available from the corresponding author on reasonable request.

## Supplementary Materials

The following supporting information can be downloaded at: https://www.mdpi.com/article/10.3390/cancers17060923/s1, Figure S1: Subgroup survival analysis of pT3 single group that comparing group 1 with only renal parenchyma invasion and group 2 with only sinus fat invasion; Figure S2: Survival analysis after excluding solely ureteral tumors with 37 cases in T3 single group and 37 cases in T3 both group; Figure S3: Subgroup analysis comparing outcomes based on pT3 single involvement in the ureter (n = 45) versus the renal pelvis (n = 19); Table S1: Clinicopathologic data of the upper tract urothelial cancer patients undergoing radical nephroureterectomy and segmental resection with central pathology review.
