# Supplementary material for: Improving Risk Stratification in pT3 Upper Tract Urothelial Carcinoma: A Focus on Invasion Patterns
Source: Cancers (Basel). 2025 Mar 8;17(6):923. doi: 10.3390/cancers17060923 (PMC11940403; doi:10.3390/cancers17060923)

Supplement Table S1: Clinicopathologic data of the upper tract urothelial cancer patients undergoing radical nephroureterectomy and segmental resection with central pathology review.

Variables	N	%
Gender		
men	168	(44.6)
women	209	(55.4)
Age		
<50	16	(4.2)
50~70	171	(45.4)
>70	190	(50.4)
Tumor location		
Renal pelvis	165	(43.8)
Ureter	128	(34.0)
Renal pelvis + Ureter	84	(22.3)
Surgical approach		
Nephroureterectomy	364	(96.6)
Segmental resection	13	(3.4)
pT stage		
pT0-1	180	(47.8)
pT2	44	(11.7)
pT3	127	(33.7)
pT4	26	(6.8)

Figure S1: Subgroup survival analysis of pT3 single group that comparing group 1 with only renal parenchyma invasion and group 2 with only sinus fat invasion.

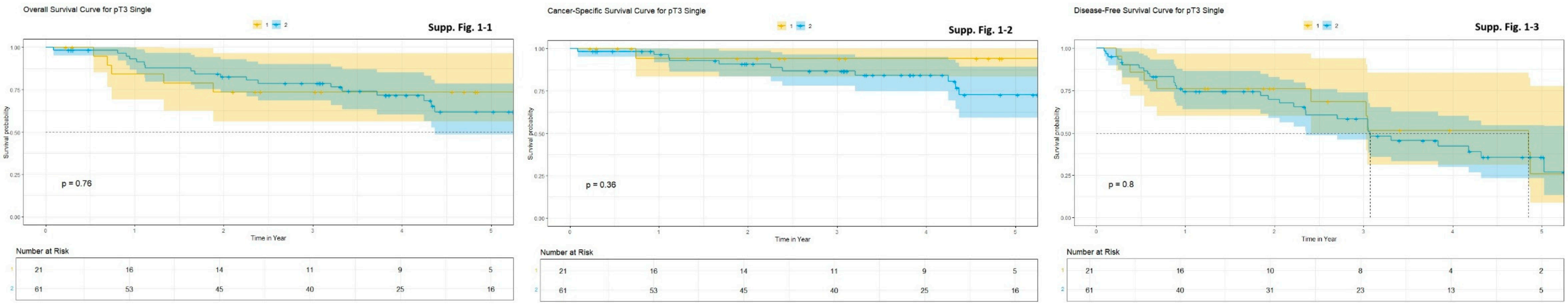

Figure S2: Survival analysis after excluding solely ureteral tumors with 37 cases in T3 single group and 37 cases in T3 both group.

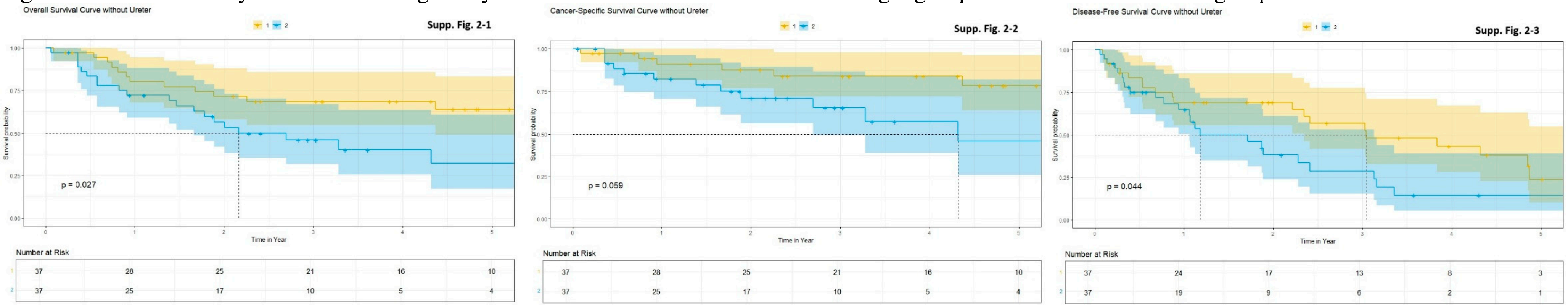

Figure S3: Subgroup analysis comparing outcomes based on pT3 single involvement in the ureter (n=45) versus the renal pelvis (n=19).

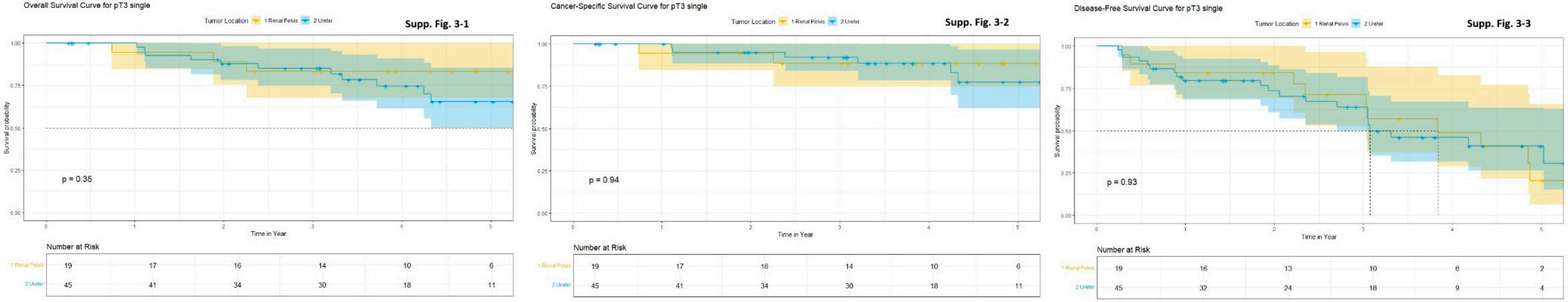

Supplement: Supplementary file 1 [file cancers-17-00923-s001.zip › cancers-3494282-supplementary.pdf]
